# Isolation and Characterization of Novel Genomic and EST-SSR Markers in *Coreoperca whiteheadi* Boulenger and Cross-Species Amplification

**DOI:** 10.3390/ijms131013203

**Published:** 2012-10-15

**Authors:** ChangXu Tian, XuFang Liang, Min Yang, HeZi Zheng, YaQi Dou, Liang Cao

**Affiliations:** College of Fisheries, Huazhong Agricultural University, Wuhan 430070, China; E-Mails: tianchangxu@hotmail.com (C.X.T.); minyangemma@163.com (M.Y.); zhengheziwansui@126.com (H.Z.Z.); douyaqi@hotmail.com (Y.Q.D.); caoliang0205@163.com (L.C.)

**Keywords:** *Coreoperca whiteheadi*, genomic-SSR, EST-SSRs, cross-species amplification

## Abstract

We described and characterized 11 expressed sequence tag (EST)-derived simple sequence repeats (SSR) and seven genomic (G)-derived SSRs in *Coreoperca whiteheadi* Boulenger. The EST-SSRs comprised 62.2% di-nucleotide repeats, 32.2% tri-nucleotide repeats and 5.5% tetra-nucleotide repeats, whereas the majority of the G-SSRs were tri-nuleotide repeats (81.4%). The number of alleles for the 18 loci ranged from 3 to 6, with a mean of 3.8 alleles per locus. The observed (Ho) and expected heterozygosities (He) values ranged from 0.375 to 1.000, and 0.477 to 0.757, respectively. The polymorphic information content (PIC) values ranged from 0.466 to 0.706. The mean values number of alleles, Ho, He, and PIC of EST-SSRs were higher than those of the G-SSRs. Four microsatellite loci deviated significantly from Hardy-Weinberg equilibrium (HWE) after Bonferroni correction and no significant deviations in linkage disequilibrium (LD) were observed. These loci are the first to be characterized in *C. whiteheadi* and should be useful in the investigation of a genetic evaluation for conservation. Compared with 11 loci in *C. whiteheadi*, 37 potential polymorphic EST-SSRs were found in *Siniperca chuatsi* (Basilewsky), which will provide a valuable tool for mapping studies and molecular breeding programs in *S. chuatsi*.

## 1. Introduction

*Coreoperca whiteheadi* Boulenger, one of the lower percoid fishes, is found in south China and the Red River of North Vietnam [1,2]. Due to anthropogenic disturbances, such as over-exploitation and environment pollution, the wild resource of *C. whiteheadi* has markedly declined in recent years [3]. Because of its dire conservation status, the genetic conservation of *C. whiteheadi* is becoming essential for the sustainable management of natural resources and increasing the production of this species. Hence, robust genetic markers are needed for information on the population dynamics of this species, including genetic connectivity, in order to inform conservation efforts.

Microsatellites or simple sequence repeats (SSRs) have become a useful marker system in population genetics analysis, genetic mapping and marker-assisted selection (MAS) of many kinds of fish species because of their co-dominant nature, high allelic polymorphism, high reproducibility and transferability across species [4,5]. However, widespread use of these markers is often limited by the time and cost involved in their development [6]. The recent development of library enrichment techniques and automated sequencing has made production of these markers simple, rapid, and cost effective [7,8]. SSR markers can be classified in genomic SSRs and EST-SSRs [9]. However, until now, no microsatellite markers have been reported for *C. whiteheadi*.

In this study, we characterized 11 EST-SSRs and seven G-SSRs as a tool to support genetic conservation and breeding programs in *C. whiteheadi*. 37 potential polymorphic EST-SSRs were also found in a sample of 12 wild *Siniperca chuatsi* (Basilewsky) individuals. A total of 122 SSRs from ESTs in our transcriptome sequencing database and 49 ones from genomic sequences of enriched libraries were selected for designing microsatellite primers and tested using PCR amplification for *C. whiteheadi*. And all the 122 microsatellites from ESTs were also tested for *S. chuatsi*.

## 2. Results and Discussion

A microsatellite-enriched library was constructed from the genomic DNA of *C. whiteheadi*. Developmental steps for the construction of the enriched library and its characteristic features are summarized in Table 1. A total of 90 putative recombinant clones were picked from the enriched library, sequenced, and analyzed for presence of SSRs. Sequence analysis revealed that 30 clones (33.33%) were redundant clones. Of the remaining 60 unique clones (66.67%), 49 (81.67% of the unique clones) were found to harbor SSR sequences (GenBank Accession number: JX449105–JX449153), and 49 can be finally used for primer design. Sequence analysis of all the SSR-containing clones indicated that tri-nucleotide SSRs were found to be more frequent (81.4%) than di-nucleotide SSRs (18.6%), and no tetra/penta/hexa nucleotide SSRs was identified in the library. Among 49 tri-nucleotide SSRs, the CCT/GGA class of repeat motif was the most frequent (50% of total tri-nucleotide microsatellites), followed by the GAG/CTC class (31%).

In this study, we characterized a set of EST-SSR and G-SSR markers as a tool to support genetic conservation in *C. whiteheadi*. A total of 37 microsatellites from ESTs in our transcriptome and 31 from genomic sequences of enriched libraries were selected successfully for designing microsatellite primers and tested using PCR amplification. 11 EST-SSRs and seven G-SSRs were found to be polymorphic, and evaluated the performance in genetic analysis using 32 individuals randomly selected from a wild population (Table 2). The number of alleles for the 18 loci ranged from 3 to 6, with a mean of 3.8 alleles per locus. The Ho and He values ranged from 0.375 to 1.000, and 0.477 to 0.757, respectively. PIC values ranged from 0.466 to 0.706 (Table 2). From the results, we could find the mean values of number of alleles, Ho, He, and PIC of EST-SSRs were higher than those of the G-SSRs. Four microsatellite loci deviated significantly from HWE (*p* < 0.0029) after Bonferroni correction (Table 2), which would be due to the limited sample size, sampling strategy and null alleles [10]. Analysis with MICROCHECKER [11] indicated that no significant LD was observed. Analysis of the nucleotide sequences of the 37 EST- and 31 G-SSRs showed that, the EST-SSRs comprised 62.2% di-nucleotide repeats, 32.3% tri-nucleotide repeats, and 5.5% tetra-nucleotide repeats. In contrast, the G-SSRs were composed mainly of tri-nucleotide repeats (81.4%).

Meanwhile, 37 potential polymorphic EST-SSRs were found in a sample of 12 wild *S. chuatsi* individuals (Table 3). Among 92 successfully amplified EST-SSRs, 37 loci (40.2% of the designed primers) showed probably polymorphism in *S. chuatsi*, compared with 11 loci (12.0% of the designed primers) in *C. whiteheadi.* The number of alleles for the 37 loci in *S. chuatsi* ranged from 3 to 8, with a mean of 4.3 alleles per locus. It is easy to explain the difference in cross-species amplification as the transcriptome in this study was from F1 interspecies hybrids between *S. chuatsi* and *S. schezeri*. The 11 loci in *C. whiteheadi* were fully contained in 37 potential polymorphic EST-SSRs in *S. chuatsi.* And the mean values of number of alleles of the 37 loci were higher than those of the 11 loci (Table 3). Only a small subset of the EST-SSRs in our transcriptome was tested in this study, but high levels of polymorphism in *S. chuatsi* indicate that the pairs of primers described here may be suitable for assessments of genetic diversity, the construction of high-density linkage map, and MAS in *S. chuatsi*, but still required further study due to the limited sample size.

## 3. Experimental Section

*De novo* transcriptome sequencing of F1 hybrids between *S. chuatsi* (♀) and *S. scherzeri* (♂) was performed and a total of 118,218 unigenes were identified. The processes of library preparation for transcriptome analysis and sequence assembly were as described in [12,13]. This unigene set was used for mining EST-SSR markers using the default parameters of the BatchPrimer3 v1.0 software [14]. The primers for these SSR loci were designed using NCBI/Primer-BLAST (Available online: http://www.ncbi.nlm.nih.gov/tools/primer-blast/index.cgi?LINK_LOC=BlastHome; accessed on 18 June 2012). In this study, a subset of 92 EST-SSR markers was screened on 12 wild *S. chuatsi* and 32 wild *C. whiteheadi*, respectively.

A microsatellite-enriched genomic library was constructed using the fast isolation by amplified fragment length polymorphism (AFLP) of sequences containing repeats (FIASCO) protocol [7]. High quality genomic DNA was fragmented using a restriction enzyme, MseI. The fragmented DNAs were ligated to specific adapters (5′-GACGATGAGTCCTGAG-3′ and 5′-TACTCAGGACTCAT-3′). The polymerase chain reaction (PCR) products were size selected to preferentially obtain small fragments (300–1000 bp), which were hybridized to one streptavidin-biotinylated oligo simple sequence repeat complexes: (CCT/GGA)_15_. The enriched DNAs were cloned into the pGEM-T vector (Promega, Madison, WI, USA) and then transformed into competent DH5a strain (Promega, USA). White colonies were randomly picked from the primary transformation plates, and then the isolated Plasmid DNA was sequenced using ABI 3730 Genetic Analyzer (Applied Biosystems, Foster City, CA, USA). Identification of SSR clones was screened using the SSRHUNTER program, which was designed to find regions containing SSRs [15]. For all types of SSRs, a minimum length criterion of 12 bp was selected, and only perfect SSR were considered. Primers flanking SSR were designed using the PRIMER PREMIER 5.0 program (PREMIER Biosoft International, Palo Alto, CA, USA). The positive clones were identified by PCR using MseI-N primers and M13 primers. Of the 90 colonies, 60 were sequenced using ABI 3730 Genetic Analyzer (Applied Biosystems), 49 of which contained microsatellites (GenBank Accession number: JX449105–JX449153). In this study, a subset of 31 G-SSR markers was screened on 32 *C. whiteheadi*, respectively.

Total genomic DNA was extracted from fin clips using the TIANamp Genomic DNA Kit (Tiangen, Beijing, China) following the manufacturer’s instructions. Polymerase chain reaction (PCR) conditions were optimized for each pair of primers. PCRs were performed in 25 μL reaction volumes containing 2.5 μL of 10 × PCR buffer, 1.0–3.0 mM MgCl_2_, 50 μM dNTPs, 0.4 μM of each primer, 1 U Taq polymerase (Takara, Dalian, China) and 50 ng genomic DNA. PCR conditions were as follows: initial denaturation at 94 °C for 3 min followed by 30 cycles at 94 °C for 30 s, the optimized annealing temperature (Table 2, 3) for 30 s, 72 °C for 30 s, and then a final extension step at 72 °C for 10 min. PCR products were separated on a 8% non-denaturing polyacrylamide gel electrophoresis and visualized by silver staining. A denatured pBR322 DNA/MspI molecular weight marker (Tiangen, Beijing, China) was used as a size standard to identify alleles.

The number of alleles (Na), the observed (Ho) and expected heterozygosities (He) were estimated using POPGENE version 1.32 [16]. The polymorphic information content (PIC) was calculated using the Formula 1:

(1) $$PIC=1-(\sum_{i=1}^{n}q_{i}^{2})-(\sum_{i=1}^{n-1}\sum_{j=i+1}^{n}2q_{i}^{2}q_{j}^{2})$$

where *n* is the number of alleles, and *q_i_*, *q_j_* is the *i*th and *j*th allele frequency, respectively [17]. Deviations from HWE and LD were tested using the online version of GENEPOP (Available online: http://genepop.curtin.edu.au/, accessed on 11 January 2011) [18]. All results were adjusted for multiple simultaneous comparisons using a sequential Bonferroni correction [19]. Genotyping errors due to null alleles, stutter bands, or allele dropout were analyzed using the software Micro-checker 2.2.3 (The University of Hull: Hull, UK, 2005).

## 4. Conclusions

In this study, 18 novel polymorphic SSR markers, 11 EST-SSRs and seven G-SSRs were successfully characterized in *C. whiteheadi*. These loci are the first to be characterized in *C. whiteheadi* and should be useful in the investigation of a genetic evaluation for conservation. Meanwhile, 37 potential polymorphic EST-SSRs were found in a sample of 12 wild *S. chuatsi* individuals, which will provide a valuable tool for mapping studies and molecular breeding programs of *S. chuatsi*.

## Figures and Tables

**Table 1:** Screening steps in the construction and characteristic features of the microsatellite-enriched library in *C. whiteheadi*.

Screening step	Number and percentage
Sequenced clones	90
Redundant clones	30 (33.33%)
Unique clones	60 (66.67%)
SSR clones	49 (81.67%)
Primer design	31
Polymorphic markers	7

**Table 2:** Characteristics of 18 polymorphic microsatellite loci in a sample of 32 *C. whiteheadi* individuals.

Locus	Gene Bank	Repeat motif	Primer sequence(5′-3′)	Na (size range, bp)	Ta (°C)	H_O_	He	PIC	P-HWE
**EST-SSR**									
CW399	JX503464	(CA)_10_N(CA)_10_	F: ATTTGCATTCGAGGTCATC	4 (104–136)	55	1.0000	0.7118	0.6464	0.4512
			R: CAGATACATCATGGTGTCCTT						
CW403	JX503460	(GT)_5_N(TG)_6_	F: CTGAATTTGAATTGTCTGGA	5 (180–263)	55	1.0000	0.7574	0.7063	0.0002 *
			R: ACACACGCTGACACTATTTC						
CW411	JX503452	(TG)_12_	F: CAACTAATCATCACAGCCCA	4 (86–116)	58	0.9688	0.7039	0.6383	0.0101
			R: GCTCCATACTGTACTCATTACCA						
CW416	JX503447	(AC)_13_N(CA)_8_(CT)_8_	F: GACCAAAGATTTCAAGGAGT	5 (185–250)	55	0.5000	0.6736	0.6155	0.3571
			R: AGTACAAAGATAGAGGGATGTG						
CW372	JX503491	(AC)_11_N(AC)_5_	F: ATCACACACTCGCAACATTC	4 (164–225)	60	0.9688	0.7257	0.6620	0.0029
			R: GTAAAGGGGTCGTATGTCGT						
CW376	JX503487	(CAG)_9_	F: CTTGGACCTGAACCTGGA	3 (250–283)	58	0.9063	0.6066	0.5299	0.9911
			R: CCACCAACACCTACTCCCTAT						
CW460	JX503403	(GT)_9_N(TC)_5_	F: AATGGGACCACCCCTCTTGT	3 (232–268)	55	1.0000	0.6344	0.5540	0.0000 *
			R: GGGGAAGGATGAGGAGTTTG						
CW426	JX503437	(GT)_13_(GA)_18_	F: TTTGAATCCTCCACTTGTA	3 (139–171)	54	0.8125	0.6652	0.5813	0.9815
			R: ATGTAGTCCGAAGCAGAAG						
CW461	JX503402	(TC)_14_	F: CGCGTCCGATCTGTTTTACC	3 (178–194)	55	0.9375	0.6647	0.5799	0.0041
			R: AAGCTGCTCTTTCATCTCGTCA						
CW745	JX503388	(TG)_18_	F: AAGAGTGCGGAAGACGAGG	4 (103–137)	55	1.0000	0.7257	0.6632	0.0120
			R: ACAACCCAACCACCACCAA						
CW467	JX503396	(TCA)_5_	F: TGGTCTCCGCACAACATCAT	5 (238–300)	55	1.0000	0.6920	0.6182	0.0000 *
			R: CCAGATCCACATCCCTTCAC						
**Mean**				3.9		0.9176	0.6874	0.6177	
**G-SSR**									
FC124	JX449121	(TCC)_6_	F: CCAACGCAGCAACATTTCTA	4 (185–222)	60	0.5313	0.6344	0.5755	0.5555
			R: TGACAAGCAGAAGAGGAGGC						
FC133	JX449126	(TCC)_9_	F: GAGTCAGCAGAAGGGAACCA	4 (101–121)	62	1.0000	0.7431	0.6829	0.0034
			R: GGGACTGGGACTAACACTTC						
FC137	JX449130	(TG)_20_	F: CCCTCTCTGTTGTCTTGGCT	6 (65–108)	62	0.6563	0.6205	0.5841	0.2640
			R: GTCTACTCCCTAACCCCAGT						
FC139	JX449132	(AGG)_5_	F: GTGCTCCAACCAGACAGAGG	4 (74–92)	60	0.9063	0.6141	0.5422	0.0003 *
			R: AACTTGTTGCTCTTCATCCA						
FC154	JX449140	(CCT)_8_	F: AGCCACGACTGTTTAGATGA	3 (197–241)	60	0.3750	0.6146	0.5242	0.0421
			R: AGAGTGCTCCAGTCCACAGT						
FC161	JX449144	(GGA)_4_	F: CAGGAGATGAGGGTGACA	3 (308–472)	60	0.4250	0.4766	0.4658	0.0261
			R: GAGGAGGGAGATACAGAAGC						
FC163	JX449146	(GAG)_6_	F: GTGTTGAAGGTGTGGAGGTG	3 (187–230)	58	0.8438	0.6592	0.5743	0.0109
			R: GTCTGTTTGGAAGGAATG						
**Mean**				3.8		0.6339	0.5804	0.5213	

Na, number of alleles; Ta, annealing temperature; Ho, observed heterozygosity; He, expected heterozygosity; PIC, polymorphic information content;

* indicates significant deviation from HWE after Bonferroni correction.

**Table 3:** Characteristics of 37 potential polymorphic EST- simple sequence repeats (SSR) loci in a sample of 12 *S. chuatsi* individuals.

Locus	Repeat motif	Primer sequence (5′-3′)		Expected size (bp)	Ta (°C)	Na	Gene Bank

		F:	R:				
CW370	(TG)_14_	CTTATTGGCTATGTGGTGCT	GTCCCTTTCTCTCTGTTTACTTC	280–380	58	5/--	JX503493
CW372	(AC)_11_N(AC)_5_	ATCACACACTCGCAACATTC	GTAAAGGGGTCGTATGTCGT	160–220	60	6/4	JX503491
CW375	(GT)_17_	TCTGTACCACCTGAGCGTTC	TCCTGTTTATGCTCCACGAC	130–160	60	4/--	JX503488
CW376	(CAG)_9_	CTTGGACCTGAACCTGGA	CCACCAACACCTACTCCCTAT	260–305	58	4/3	JX503487
CW379	(TC)_12_N(CT)_5_	GGTCATACCTGGTGTAGTCACT	ATTACCCAGAAGCACTAGCAT	170–212	58	4/--	JX503484
CW380	(CCA)_6_N(TCA)_7_	CCTCTGCGCTAACTAACA	CTTCTCCAGCTTCTTCAAAC	147–201	55	4/--	JX503483
CW382	(AC)_5_N(TG)_14_N(GT)_5_	GAAGCAACACAGCCAGAAGAGG	TTCCACGGCGTCTCCAAC	350–410	60	3/--	JX503481
CW389	(AC)_22_N(AC)_5_N(TG)_5_	TAACAGACAAAACCTGGACT	ATGGCTCCTGGTATTTAGTG	280–350	58	5/--	JX503474
CW390	(AC)_12_	CAACAATGAGAACCCACAGA	CAGCAAAGAGCAGAGTTGTA	217–280	58	5/--	JX503473
CW392	(AAT)_5_	GATAATACTGCTGAGCAAGTC	TCATCACACTCAGTAGCAAC	220–310	58	3/--	JX503471
CW395	(CA)_11_	CACAGAAGTGAACCGACA	CAACAGTCTTGACAATCTCTCT	200–250	55	5/--	JX503468
CW398	(CA)_6_N(CA)_14_N(CA)_5_	ACAGTTTGACGGAGTGTAAC	ACTTCAGACAAGCAAATGTG	147–230	58	5/--	JX503465
CW399	(CA)_10_N(CA)_10_	ATTTGCATTCGAGGTCATC	CAGATACATCATGGTGTCCTT	90–155	55	5/4	JX503464
CW403	(GT)_5_N(TG)_6_	CTGAATTTGAATTGTCTGGA	ACACACGCTGACACTATTTC	190–260	55	4/5	JX503460
CW408	(ATC)_11_	GCATTAGGGACATTAGGTACTC	GCCATGTTTCAGTTCAGGTA	125–210	58	4/--	JX503455
CW411	(TG)_12_	CAACTAATCATCACAGCCCA	GCTCCATACTGTACTCATTACCA	100–171	58	5/4	JX503452
CW412	(AC)_18_	ACATCGAGACTACTGGAAGG	CATCAATTACTTGGTCTCCAT	170–210	55	4/--	JX503451
CW413	(TTGT)_5_	AGGTGTGAACTATAAGTAGCACG	ACTCCACTGGCTACACACAT	123–170	60	3/--	JX503450
CW416	(AC)_13_N(CA)_8_(CT)_8_	GACCAAAGATTTCAAGGAGT	AGTACAAAGATAGAGGGATGTG	180–210	55	4/5	JX503447
CW425	(TCT)_6_	TCATAGCTCCGCCTACACCA	CAGCGACGCACCAGCAAAA	170–300	56	5/--	JX503438
CW426	(GT)_13_(GA)_18_	TTTGAATCCTCCACTTGTA	ATGTAGTCCGAAGCAGAAG	130–255	54	8/3	JX503437
CW429	(CA)_20_	TCACTAAGATGATTGCTGC	TGACTACTGTTGTGATGGG	200–310	54	5/--	JX503434
CW432	(GT)_20_	ATATTCCAGTGGGTTTTACTTACA	GCTAGCCTCCCTCCCTTA	200–250	57	4/--	JX503431
CW437	(AATA)_5_	CACATATTAACAAGTCAGCGTGAG	ATGGCTTTGAGTTCTGAGACGA	210–270	55	4/--	JX503426
CW443	(TGA)_8_	AACTGTTGGTGGTGATGAGGG	GATCGTGTTGGAAAGAATGTCG	160–220	55	5/--	JX503420
CW447	(AC)_12_	GCTGGTTCTGGCTCTGGTC	TATAAGGAATGTGAGGGTCTGG	200–280	57	3/--	JX503416
CW454	(TG)_13_	ATCCATCCAGGTTCAAGTGC	TCAATCTTCTCTTTATTGACCACAT	280–355	55	5/--	JX503409
CW457	(TTA)_5_N(TG)_6_	CTGACTCATGCTGCATATTGTGA	GCCAGGGTGCAATTAAGTGT	242–309	55	4/--	JX503406
CW460	(GT)_9_N(TC)_5_	AATGGGACCACCCCTCTTGT	GGGGAAGGATGAGGAGTTTG	210–260	55	3/3	JX503403
CW461	(TC)_14_	CGCGTCCGATCTGTTTTACC	AAGCTGCTCTTTCATCTCGTCA	170–200	55	3/3	JX503402
CW465	(CTG)_7_	CTGACAGGCAGAAGGTAGCA	ATTTAGCAGAGCTTTGACCC	180–280	55	4/--	JX503398
CW467	(TCA)_5_	TGGTCTCCGCACAACATCAT	CCAGATCCACATCCCTTCAC	220–290	55	5/5	JX503396
CW740	(GT)_9_T(TG)_12_	AGACATCCCAATGTGGCAGAA	ACGCAAGACAAGCCATACGTG	210–300	55	4/--	JX503393
CW745	(TG)_18_	AAGAGTGCGGAAGACGAGG	ACAACCCAACCACCACCAA	107–160	55	4/4	JX503388
CW754	(AC)_23_	AGAATGGTCAAACTCCTGCAA	ATCACTATTTACCACGTACACTCGA	160–275	55	6/--	JX503379
CW757	(ACC)_7_	CTAGGACGCTTCCAAACTGG	GGGAGAAAGGGTAGCAGAGG	250–300	58	4/--	JX503376
CW759	(CA)_13_N(CA)_8_	TGGGCGACGGCTTAGAAAG	TATCGAGTGAATCATTGGAATCG	250–330	54	3/--	JX503374
**Mean**						4.3/3.8	

For each locus the information of number of alleles, in the left refers to *S. chuatsi* and the right refers to *C. whiteheadi. Ta* corresponds to annealing temperature; *Na* is number of alleles; no polymorphism for each locus is denoted by “--”.
